# Bacterial nanocellulose stimulates mesenchymal stem cell expansion and formation of stable collagen-I networks as a novel biomaterial in tissue engineering

**DOI:** 10.1038/s41598-018-27760-z

**Published:** 2018-06-20

**Authors:** Martin Vielreicher, Dana Kralisch, Simon Völkl, Fabian Sternal, Andreas Arkudas, Oliver Friedrich

**Affiliations:** 1 Institute of Medical Biotechnology, Department of Chemical and Biological Engineering, Friedrich-Alexander University of Erlangen-Nürnberg, Paul-Gordan-Str. 3, Erlangen, 91052 Germany; 2 Institute of Pharmaceutical Technology. Faculty of Biology and Pharmacy, Friedrich-Schiller-University Jena, Lessingstr. 8, Jena, 07743 Germany; 3 Department of Internal Medicine 5, Hematology and Oncology, University Hospital Erlangen, Ulmenweg 18, 91054 Erlangen, Germany; 4 Department of Plastic and Hand Surgery, University Hospital Erlangen, Friedrich Alexander University of Erlangen–Nürnberg, Krankenhausstr. 12, 91054 Erlangen, Germany

## Abstract

Biomimetic scaffolds are of great interest to tissue engineering (TE) and tissue repair as they support important cell functions. Scaffold coating with soluble collagen-I has been used to achieve better tissue integration in orthopaedy, however, as collagen persistence was only temporary such efforts were limited. Adequate coverage with cell-derived ECM collagen-I would promise great success, in particular for TE of mechanically challenged tissues. Here, we have used label-free, non-invasive multiphoton microscopy (MPM) to characterise bacterial nanocellulose (BNC) - a promising biomaterial for bone TE - and their potency to stimulate collagen-I formation by mesenchymal stem cells (MSCs). BNC fleeces were investigated by Second Harmonic Generation (SHG) imaging and by their characteristic autofluorescence (AF) pattern, here described for the first time. Seeded MSCs adhered fast, tight and very stable, grew to multilayers and formed characteristic, wide-spread and long-lasting collagen-I. MSCs used micron-sized lacunae and cracks on the BNC surface as cell niches. Detailed analysis using a collagen-I specific binding protein revealed a highly ordered collagen network structure at the cell-material interface. In addition, we have evidence that BNC is able to stimulate MSCs towards osteogenic differentiation. These findings offer new options for the development of engineered tissue constructs based on BNC.

## Introduction

In tissue engineering (TE), usually MSCs^1,2^ are seeded and expanded on cyto-compatible, biomaterial scaffolds to ensure a physiological cellular environment^3,4^. MSCs are multipotent cells which can differentiate into numerous cell types including bone, cartilage, muscle, fat and connective tissue cells^2,5^. One of the requirements for a scaffold is to provide structural support for cell anchorage and subsequent 3D tissue formation^6^. In load-bearing tissues (e.g. bone, skin, tendon and ligament), cell organisation and stabilisation is mostly provided by extracellular collagen-I fibre networks^7–9^. The engineering of such tissues thus, requires suitable conditions which favour and support the formation of collagen-I networks^4,10^.

BNC is a novel and highly interesting advanced biomimetic material^11–13^ which was studied in various contexts^14^, regarding scale-up of production^15^, bio-composite development^16^, use as implant^12,17–19^ or wound dressing material^11,20^ and drug release^21,22^. It is biotechnologically produced and can be arranged into mechanically stable semi-transparent hydropolymer fleeces. The natural origin and nano-fibrillar and micro-porous composition renders it interesting for use in TE applications (e.g.^23–25^).

Unlike traditional methods to quantify extracellular matrix (ECM) production that do not provide any details of the steric arrangement and quality of produced ECM (i.e. Western blot detection), we were particularly interested in developing *in situ* imaging approaches to visualise ECM production in 3D. MPM of cell-seeded constructs can overcome some of the constraints of conventional microscopic imaging (i.e. invasiveness/destructiveness and limited penetration depth; as reviewed in^26^) very elegantly by imaging to several hundreds of µm deep within artificial tissue^27^ without compromising tissue integrity by bleaching or labelling artifacts. A special non-linear case of MPM, SHG microscopy, is able to specifically image the formed collagen-I fibre networks^28–30^ with minimum scattering due to very confined excitation in the µm³ range, and use of near-infrared fs-pulsed laser light^31–33^. Collagen-I is one of only a few biomolecules that is capable of emitting SHG light due to its non-centrosymmetric structure, however a higher assembly grade (fibre bundling) is required. By recording cellular autofluorescence (AF) derived from nicotinamide adenine dinucleotide (NAD) and flavin molecules in parallel, a more thorough examination of cell behaviour can be monitored during culture, especially when combined with labelling experiments^34^.

In this study, we tested BNC and its potency to support MSCs to form collagen-I fibrous networks, which we detected with SHG. We were in particular interested in the efficiency and quality of collagen-I formation from cell type, media composition (serum, ascorbic acid) and cell architecture perspectives (2D versus 3D culture)^35,36^. In this regard, a nano-fibrous material forming micro-pores on the surface, like BNC, was of interest to understand how 3D cell distributions may support enhanced formation of collagen networks for higher stability of engineered tissue. We also tested the AF and SHG on the surface and within. After cell seeding we studied collagen-I and cell multilayer formation. BNC showed distinct MPM signal patterns, enabling the analysis of material surface and inner structure as well as cell-material interface. Its nano-fibrous and micro-structured surface landscape stimulated the cells for fast and almost entire population, strong proliferation and ECM production. Cavity-like structures present on the fleeces appeared to effectively stimulate cells to form multilayered cell arrangements and collagen-I matrix formation.

In TE, biocompatible materials which support cell attachment and stimulation, and which deliver micro-environments for the cells are urgently needed. BNC enables MSCs to form their own native collagen-I matrix. This provides stability for the MSCs. As collagens are highly conserved between species, the laid-down collagen networks can also act as templates for cells from different origin. The findings are therefore important to the field of TE and regenerative medicine, but also highly interesting for the development of clinical products^37^ and potential use as model platforms for scientific investigation in life sciences.

## Materials and Methods

### Cell Culture

We used MSCs isolated from rat femur bone marrow in the laboratory of co-author A.A. and were surplus from another unrelated study^38^. Their isolation was based on cell adherence to cell culture plastic surfaces and their osteogenic differentiation capacity as verified by staining for alkaline phosphatase after 10–14 days in differentiation medium. Cell selection criteria were as follows (as also described in^39^): positive for CD90, CD29, CD54 and CD73 and <5% positives for CD45 and CD31 surface markers. As no animals were sacrificed for this study ethical approval was not required.

In our experiments, MSCs from passage number six were used. The cells exhibited good growth (doubling time of ~24 h) and morphology and were cultured in polystyrene flasks (tissue-culture grade, T-75 and T-25 format; Sarstedt, Germany) for a maximum of three weeks for each test. Cells were then washed with PBS, detached using 0.25% trypsin/EDTA solution (Biochrom, Germany) for 3 min and centrifuged at 100 g for 3 min. Cell counting was performed with a haemocytometer, the living cell fraction was determined using trypan blue. The MSC basal medium was DMEM-Ham´s F12 (#21331; Thermo Fisher, USA) which was enriched with 2 mM L-glutamine (Biochrom). The L929 cell line (mouse connective tissue fibroblasts, No. ACC2, DSMZ, Germany) was chosen as a reference^40^. Their basal medium was RPMI (#11875, Life Tech) which already contained L-glutamine. Both basal media were free of ascorbic acid and supplemented with penicillin-streptomycin. MSC standard medium (Std) contained 20% and L929 Std medium 10% FBS (Superior grade, #S0615, Biochrom). Stimulation media were reduced in serum to 1% and 3% FBS and supplemented with L-ascorbic acid (aa, #A4544; Sigma-Aldrich, Germany) to 25 µg ml^−1^ (1x) or 100 µg ml^−1^ (4x). Cell media were exchanged every two to three days.

### Bacterial Nanocellulose

BNC was produced by the *Komagataeibacter* x*ylinus* strain DSM 14666. The biomimetic material was synthesised by static cultivation (~7 days) in Hestrin-Schramm media^41^ at the interface between air and culture medium resulting in fleeces with a layered structure^42^. 1 L culture medium was obtained from 20.00 g D-glucose (anhydrous, Carl Roth, Germany), 5.00 g Bacto yeast extract (Difco, BD Biosciences, USA), 5.00 g Bacto peptone (Difco), 3.40 g Disodium hydrogen phosphate * 2 H_2_O (Carl Roth), 1.15 g citric acid * H_2_O (Carl Roth) diluted in deionised water. The mixture was stirred until the solution was clear. Prior use, the culture medium was sterilised via autoclaving (121 °C, 20 min). The fleeces were purified by treating with boiling 0.1 N sodium hydroxide solution for 30 min and by rinsing with deionised water until a neutral pH-value was observed. Afterwards, excess water was removed by low vertical pressure. The basic units are characteristic cellulose micro-fibrils of uniform thickness and low fibre length variations (low polydispersity index). Intermolecular hydrogen bonds enable the formation of fibre bundles which organise into anisotropic networks that vary in horizontal and vertical direction. The average pore size is 1 × 3.5 µm with low size variations. Cellulose fibre diameters are in the nm range (5–20 nm). The material consists of both amorphous and crystalline regions, but in the hydrated state, the overall degree of crystallinity is relatively high^15^. Native BNC has a very high water adsorption capacity (WAC = m_wet_/m_dry_ = 120, water content <99%) and is almost transparent. BNC has also a relatively high tensile strength of 0.5–0.6 MPa (measured for dumbbell-shaped BNC fleeces in never-dried state^43^) and can be thermally sterilised. The compressive strength value is 0.19 ± 0.01 MPa^43^. The biomaterial shows good biocompatibility, is very skin-friendly and can be used as a wound dressing material. Due to its good protein loading and release profiles BNC may act as carrier material for bioactive substances^21,22^. BNC was provided both as 50 × 50 × 2 mm layers and as freeze-dried rolled sheets (thickness: 0.2 mm). Hydrated BNC was cut into ~8 × 8 mm patches and equilibrated in the respective growth medium overnight at 37 °C. For cell seeding, the medium was removed and suspensions of cells were added on top. BNC was kept in the cell incubator until a cell density of ~90% was reached after 1–2 days, then stimulation media were added or cells remained in Std medium. As patches were partially transparent, cell coverage was followed by trans-illumination microscopy. For imaging, patches had to be flipped-over to access the cells from below (especially for MPM).

### Phase Contrast Imaging

A patch of BNC fleece was positioned upside-down on glass cover slips and imaged with an inverted microscope (Eclipse Ti, Nikon, Japan) using a 10x water immersion objective (CFI Planfluor Ph1 DLL, N.A.: = 0.3, Nikon) and transmission white light. Detection occurred with a vacuum-cooled sCMOS camera (Neo 5.5, Andor Technology, Northern Ireland).

## Fluorescence Labelling and Microscopy

### (A) Collagen-I labelling with CNA35-AlexaFluor546

For direct imaging of collagen fibres, CNA35 (collagen adhesin, protein data base accession number: 2F68), a collagen-I binding protein domain from *Staphylococcus aureus*^44^ was used (kindly provided by Magnus Hook/Brooke Hageman-Russell, Texas A&M Health Science Center, USA). CNA35 protein was labelled and purified with Alexa Fluor 546 Protein Labeling Kit (A10237, Molecular Probes, USA). The Alexa Fluor 546 reactive dye has a succinimidyl ester moiety that reacts efficiently with primary amines of proteins to form stable dye–protein conjugates. The conjugate (CNA35-AF546) was concentrated using Amicon Ultra-4 10 K centrifugation filter units (Merck Millipore, USA) and tested for purity and degree of labelling using protein electrophoresis (SDS-PAGE) and a UV-Vis spectrophotometer (NanoDrop, Thermo Fisher). MSCs were grown to confluence on BNC in standard growth medium before staining with CNA35-AF546 occurred. Imaging was performed on a SP3 confocal spectral microscope (Leica Microsystems, Germany) equipped with a TCS SL scanner using a He-Ne laser (λ = 543 nm) for excitation and, alternatively, on an inverted epi-fluorescence microscope (Eclipse Ti, Nikon) with a TRITC filter set and vacuum-cooled sCMOS camera (Neo 5.5, Andor Technology). Excitation light was delivered by a Xenon lamp (Lambda DG-4, Sutter instruments, USA).

### (B) Actin cytoskeleton staining

For imaging structures at the cell-material interface, MSCs (2 × 10^6^ cells) were seeded on BNC patches pre-equilibrated with medium overnight and cultured. After three days, cells were washed 3x with PBS, fixed with 4% PFA for 10 min, washed again (3x) and permeabilised with 0.1% Triton X-100 for 3 min and washed 3x followed by co-labelling with phalloidin-PF546 (PromoKine, Germany) and DAPI (nucleus). For imaging, the Eclipse Ti fluorescence microscope (A) with a TRITC/DAPI filter set and 60x objective (Plan Apo VC WI DIC N2) was used. Cells were imaged from below (inverse microscope).

### Label-free Multiphoton Microscopy

For imaging, BNC patches were transferred (cells at the bottom) to 35 mm glass bottom dishes (#D35–20–1-N, Cellvis, USA) with thin cover slip (170 µm) which allows 2-photon excitation. MPM was performed on a TriMScope II, a high-performance multifocal multiphoton microscope (LaVision BioTec, Germany) based on a Nikon Eclipse Ti-E inverted stage and equipped with an ultra-short femtosecond-pulsed Ti:Sa laser (Chameleon Vision II, Coherent, USA) with dispersion pre-compensation and tunable wavelengths in the range of λ = 710–980 nm (pulse duration: t < 150 fs, repetition rate: 80 MHz). Images were collected using a water immersion LD C-apochromat objective (40 × /N.A.:1.1/UV-vis-IR/WD: 0.62; Zeiss, Germany) and detected with two high-sensitivity GaAsP photomultipliers (H7422-40, Hamamatsu Photonics, Japan), mounted in non-descanned configuration close to the back aperture of the objective. In all experiments, 2-photon excitation occurred at λ = 810 nm. Signals were separated by a dichroic mirror (460DCXR, Chroma Technology, USA). Wavelengths above 460 nm were detected in channel 1 (cellular AF), shorter wavelengths were reflected and blocked with a 405/20 bandpass filter (ET405/20x, Chroma) to image SHG in channel 2 (mainly collagen-I, SHG in backscattering mode). Alternatively, AF and SHG signals were detected unseparated in channel 1 (labelled in Figures as “AF + SHG”). Other than for AF, detection of SHG from collagen-I by cells required higher laser powers (P = ~30 mW vs 15–20 mW) at the sample position as determined with a power meter. Image overlays (AF/SHG), size determinations and mean intensity calculations (using the Analyze/Histogram function) were performed using the software Fiji.

### Fibre Orientation Analysis

Orientation analysis of collagen networks was performed with Orientation J, an Image J Plugin as previously described^40,45^. Orientation J enables the automated 2D orientation analysis of local structure elements like fibres. In Orientation J, a structure tensor is determined for every pixel based on Gaussian gradient values of pixels within the local neighbourhood. The size of the neighbourhood is specified by a Gaussian window. By means of the structure tensor an orientation and coherency value is calculated for every pixel. The orientation angle [−90°; +90°] is then visualised color-coded as colour survey image. The coherency value is a measure for the isotropy of the gradient values within the local neighbourhood and visualises the colour saturation in the colour survey image. The distribution of orientation incidents are mapped as histogram (S-distribution) which is weighted by the coherency. The histogram considers only pixels with values higher than the defined energy and coherency threshold values. The selected pixels are visualised as S-colour survey image.

### Flow Cytometry

The differentiation status of MSCs was analysed with flow cytometry two days after starting culture in Std medium (day 0) and after 16 days culture on 10 × 10 mm BNC patches (2 × 10^6^ cells) versus T-75 standard cell culture flask. Trans-illumination microscopy showed an extremely large number of cells after 16 days on the BNC fleeces. Sufficient cell numbers could be detached only after prolonged washing (>1 h, falcon tube on roller shaker) with serum-free basal medium due to serum components stored within the biomaterial inhibiting the action of trypsin. In addition, the cells anchored very strongly within the cracks on the rough BNC surface which required prolonged trypsin treatment (5–6 min). After detachment, MSCs were resuspended in full medium and sieved with a cell strainer (40 µm mesh size). MSCs were counted and checked for the living cell faction (trypan blue staining) and 10^5^ cell were stained in 1:200 dilutions with anti-rat CD31 (polyclonal, R&D systems, USA), CD34 (polyclonal, Bioss antibodies, USA), CD73 (clone 5 F/B9, BD Biosciences), CD90 (clone OX-7, BD Biosciences) and CD45 (clone OX1, eBioscience) antibodies. Antibody-positive controls were not performed as all antibodies were routinely tested and used in the laboratory. Isotype control antibodies were used as negative controls to determine non-specific background signal. Data acquisition was performed on a FACS LSR Fortessa (BD Biosciences). Viable MSCs were determined by forward scatter- (FSC) and side scatter-A, doublets were excluded by FSC-H. Scatter results showed that the MSCs used for the experiment were a very homogenous population in size and granularity with an overall low degree of granularity which confirmed our observations with microscopy. The data were analysed with FlowJo v10 (FlowJo, LLC, USA), Y-axis of histogram overlays was normalised to mode.

### Alkaline Phosphatase (AP) Assay

MSCs (1.5 × 10^6^ cells) in passage 6 were seeded to BNC patches pre-equilibrated in full medium (Std) or on normal T-25 cell culture dishes, grown in Std medium and harvested at days 0, 4, 7, 10 and 14. MG-63 human osteosarcoma cells constitutively expressing AP were used as controls (kindly provided by Rainer Detsch, Institute of Biomaterials, FAU Erlangen-Nürnberg). Thawed MG-63 cells were cultured for 1 week to build up AP activity. For detachment of MSCs from BNC, it was required to incubate the patches for at least 1 h in DMEM-F12 basal medium (50 ml falcon tube, roller shaker) to wash out serum which effectively inhibited the action of trypsin. All harvested cell samples were washed 2x in PBS to remove remaining serum components interfering with the assay. Two hundred thousand cells were lysed in 500 µl lysis buffer (TBS + 25 mM Tris-HCl, pH 8.5 + 0.5% Triton X-100) for 10 min on ice. Repeated freezing (3×, N_2(l)_) and thawing was applied to ensure completion of lysis. Cell debris was removed by centrifugation (16,000 g, 1 min, 4 °C). The AP activity test was carried out using CSPD/Sapphire-II chemiluminescent substrate (Tropix, Applied Biosystems, USA). Lysates (20 µl) were incubated with 100 µl CSPD substrate in non-transparent multi-well plates (Costar 96-well) for 20 mins at RT. Signal counts were detected after 20 mins in Luminometry mode (measurement time: 1 s) on a Victor X4 Multimode Plate Reader (Perkin Elmer, USA). Lysis buffer without cells plus CSPD substrate was taken as negative control. Counts were analysed with WorkOut 2.5 software.

### Statistical Analysis

Statistical analysis of the AP assay was performed using the nparLD package described in^46^ within the statistical computing environment of R Studio (v.3.3.3; R Core Team 2017, R Foundation, Vienna, Austria). The approach constitutes a non-parametric method tailored for assessing longitudinal data from factorial experiments displaying a repeated measurement design. The package summarises different non-parametric methods for the most frequent factorial designs, provides estimators of relative treatment effects (RTEs) and tests the hypotheses of ‘no treatment effect’, ‘no time effect’ and ‘no interaction between treatment and time’. The non-parametric statistical tests utilise ANOVA-type statistics modified for small sample sizes and further refined to consecutively investigate effects within the treatment factor over time. Further methodological details can be found in^47^.

### Data Availability

The datasets generated during and/or analysed during the current study are available from the corresponding author on reasonable request.

## Results

### New structural and optical properties of BNC

**Figure 1:**
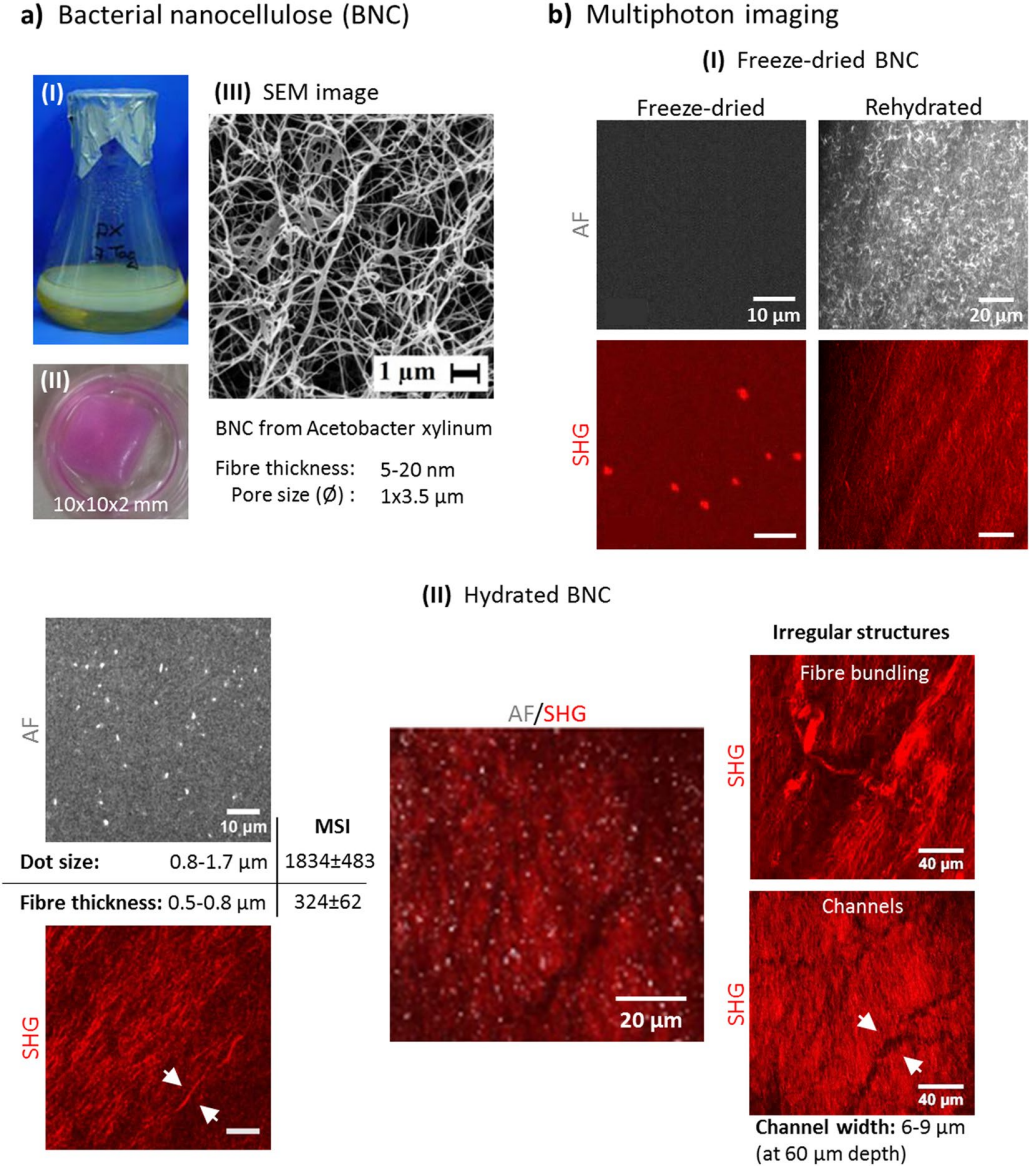
BNC characterisation. (**a**) BNC biomaterial is formed as fleeces during static culture at the air-liquid interface (I). BNC fleece patch used for cell seeding (II). (III) SEM image (magnification M = 3,000x)^22^ providing insight into the nanocellulose network ultra-structure. Images I and III were provided by D. Kralisch. (**b**) Multiphoton pattern of dried BNC. The pattern of rolled BNC rehydrated with PBS dramatically changes within minutes. (**c**) Simultaneous AF/SHG images from within the BNC exhibit both dot- to elliptic-shaped AF signals and fibre-shaped SHG signals with different signal strengths (MSI, mean signal intensity). Representative overlay image shown in the middle. Irregular structures within the cellulose network were observed as well.

**Figure 2:**
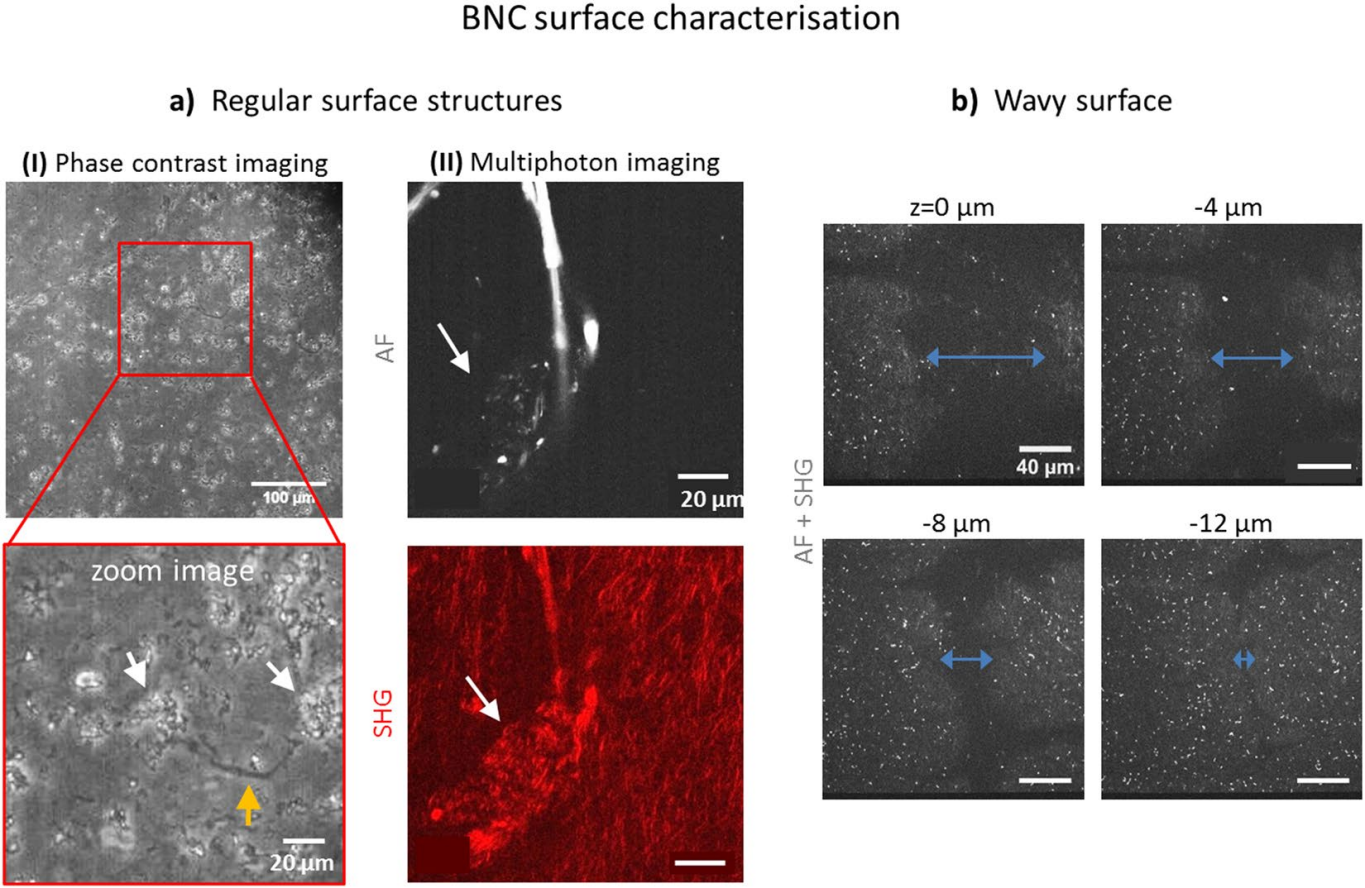
Micro-structure of the BNC surface. In (**a**), regular surface structures are presented. (I) Phase contrast images (M = 10x) reveal structures reminding of cracks (orange arrow) and cavities (~10–20 µm in diameter, white arrows) all over the surface. In (II), a typical structural islet with an agglomerate multiphoton pattern is shown (white arrows in AF and SHG images). In (**b**), a trench is displayed in various depths which is large enough to act as cell seeding core (diameter of > 60 µm at surface, unseparated multiphoton image, M = 40x).

As a new kind of natural polymer and potential scaffold material, BNC was provided as mechanically stable hydropolymer fleeces (Fig. 1a,I-III). BNC exhibits interesting biomechanical features similar to certain biological tissues (see Materials and Methods section)^42^ and is known to consist of networks of nano-sized uniform fibre arrangements and pores in the low µm range (SEM image in III). The internal structure is made up of cellulose fibres oriented in parallel. From previous work, it was known that cellulose fibres are susceptible to SHG due to their non-centrosymmetric macromolecular structure, high assembly grade and crystallinity^48^. We were interested in the optical properties of freeze-dried BNC (rolled) and its rehydration behaviour (Fig. 1b-I). This material had a very low thickness as the hydrated BNC (thickness: 2 mm) consists of less than 1% of cellulose (>99% water). No intrinsic AF, but large dot-shaped SHG signals were observed here. Rehydration however, rapidly resulted in a curly/wavy AF signal pattern and a clearly different fibre-shaped finely structured SHG pattern. During ongoing hydration, the AF pattern only slowly developed into the dot-shaped signals as seen in hydrated BNC (Fig. 1b-II, see also Supplementary Video 1 in the Supporting Information). Unexpectedly, hydrated BNC, purified by a standard method often described in the literature, exhibited a clear AF signal pattern with evenly distributed punctuate to ellipsoid structures (mean diameter: 0.8–1.7 µm). This is a finding that, to our knowledge, has not been reported before and deserves future investigation. SHG imaging revealed the sub-µm fibre structure of BNC (0.5–0.8 µm). The mean signal intensity (MSI) of AF signals was ~6x higher than SHG signals as determined with identical excitation and detection settings and calculation from multi-stack images using Fiji software (compare to Video 1). The autofluorescent dots served as effective landmarks for detecting the material border, especially when studying the cell-material interface. The dot pattern could be detected up to a depth of ~450 µm within the material which is according to findings from previous studies^49,50^. BNC showed variations in the regular distribution and appearance of AF and SHG signals with interruptions in the sheet structure, regions of more chaotic fibre distribution and bundles of thicker fibres (top right and Video 2). Deep within the material, wide-spread channel systems were detected which may act in liquid transport and exchange (bottom right). In addition, large spots with very bright AF intensities have been detected in structural anomalous regions or zones (e.g. Fig. 2a-II).

### BNC exhibits characteristic micro-structured surface domains

Phase contrast imaging revealed that the surface of hydrated BNC is not even but to some extent wavily (Fig. 2a-I). The overview image uncovers large trenches as well as elevated areas and shows that the material exhibits a high density and a certain regularity of those naturally grown structures. They can be explained by a variation in the density of the bacteria population at certain areas of the growing BNC fleece. The zoom image below furthermore reveals that both cracks (orange arrows) are present and light-diffracting cavity structures (white arrows) with >20 µm in size are evenly distributed on the surface. Unfortunately, it is not possible to perform 3D rendering of these structures for higher resolution as multiple imaging planes contribute to phase contrast transmission images. However, valuable information came from separate multiphoton images (AF/SHG, Fig. 2a-II) which in addition revealed abnormal signal patterns in both channels reminding of cavities. In the AF image, a typical structure with elliptic shape is displayed in which AF spots of various sizes with dramatically increased fluorescence intensity were observed. SHG signals co-localise to these structures determining their size to ~30–40 µm. Compared to the typical regular sheet-like SHG fibre pattern, a clearly differing agglomerate wavy fibre pattern becomes obvious. Their dimensions resemble the cavities presented in (I). We have named these areas ‘structural islets’ (for a 3D representation see Supplementary Video 3). Next, we were interested in investigating the surface topography of BNC. A series of multiphoton images taken at increasing depth (in steps of 4 µm beginning at the surface) is presented in Fig. 2b. A large representative trench of more than 60 µm in diameter at the BNC surface is displayed which narrows to <10 µm at a depth of 12 µm. Such structures provide ideal spatial conditions as potential cell seeding cavities and show that the material is highly uneven at the surface.

### Adhesion and organised growth of MSCs on BNC

**Figure 3:**
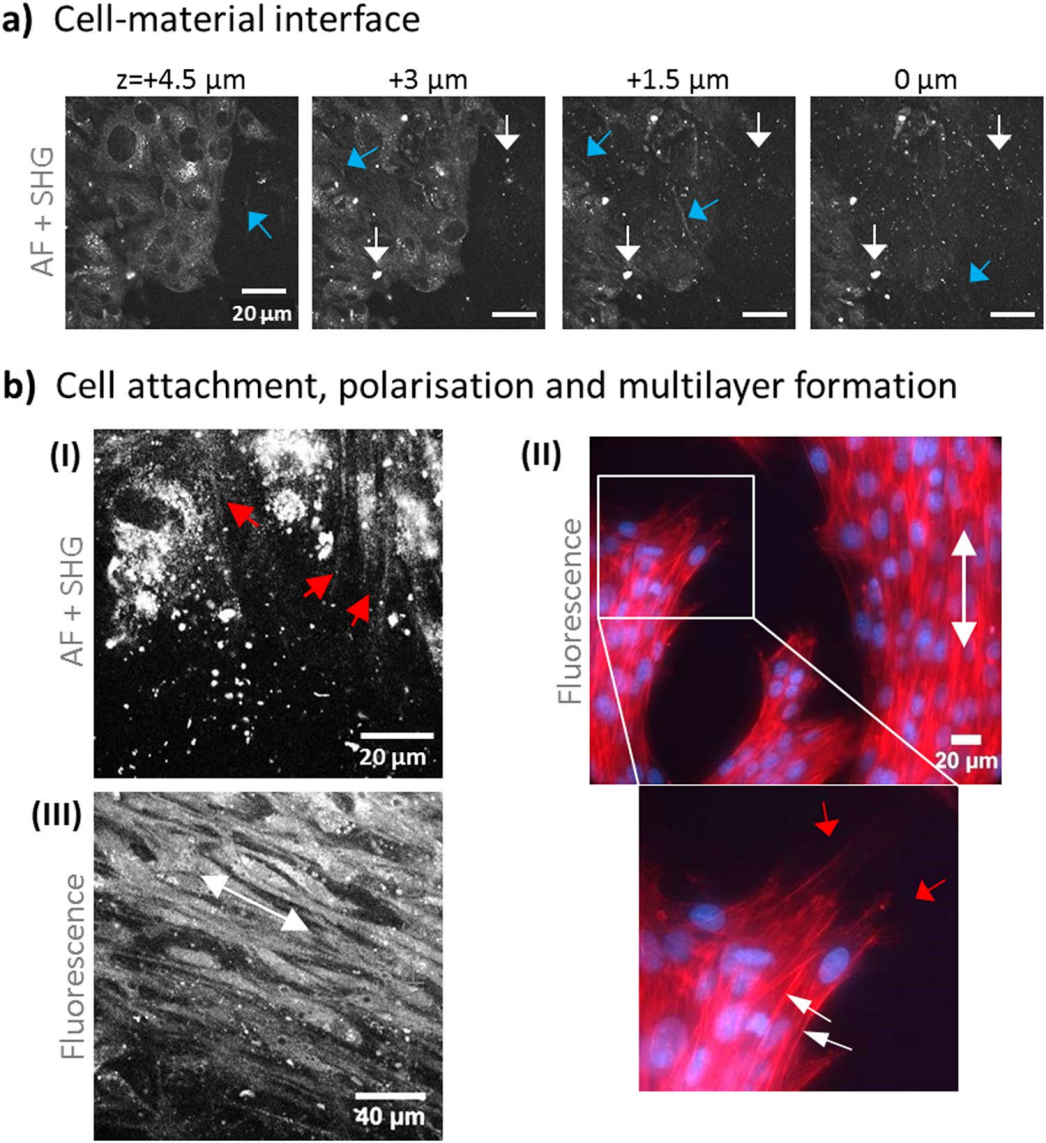
Cell-material interface: attachment, anchoring and orientation on BNC. In (**a**), multiphoton images (unseparated, AF + SHG) highlight the MSC attachment site and a few µm above. Signals derived from BNC are visible as dots (white arrows) and fibrous structures (blue arrows). The cells become visible by their AF signal. In (**b**), cell anchoring structures (filopodia) are shown in both an MPM (I, red arrows) and a fluorescence image (II, phalloidin-PF546 and DAPI). The zoom image in (II) highlights anchoring structures (red arrows) and multilayer cell arrangements (white arrows directed to DAPI-stained nuclei). Cell orientation patterns (white arrow) were observed in (II) and in (III) in between cell layers (staining with Calcein AM).

Apart from structural aspects of BNC, our major focus was on how MSCs would interact with the material. MSCs isolated from rat femur marrow^40^ have been selected because of their potential to develop into multiple terminally differentiated cell lines, including keratinocytes, osteoblasts, chondrocytes, myocytes or fat cells, rendering them highly interesting for TE of various tissues and organs. Additionally, MSCs are well known to form their own ECM and to attach well to surfaces forming distinct filopodia extensions. MSCs were seeded on BNC to test the biomaterials properties of supporting cell attachment, anchoring and growth (images from focal plane at the cell-material interface, Fig. 3). The cells adhered and spread out very fast and homogenous and covered the material surface almost completely. Figure 3a shows a series of unseparated multiphoton images from the cell layer down to the material surface. At + 4.5 µm, the cell layer is clearly visible by cellular AF, but the typical BNC fibre structures (SHG, blue arrows) and dot structures (AF, white arrows) were already visible even in between cells. At 0 µm (surface), parts of the cell layer are still in the focal plane (bottom left of the image) while other regions already show signal coming from nanocellulose fibres. Such step-wise disappearance of cells and appearance of material structure points towards some degree of unevenness of the surface in the µm regime. Video 4 presents the complete sequence of 16 images in high resolution. Figure 3b highlights additional aspects and details of cell-BNC interaction. Structures extending from the cells become visible in I (multiphoton images). Their morphology and location relative to the cells suggest that these are filopodia. Below in the image, dotted AF signals from BNC become visible. Such autofluoresent structures are also partially visible inside the cells, however, these can be differentiated well due to their pattern close to the nucleus appearing almost in black. To independently highlight anchoring structures at the cell-material interface, actin-/DAPI staining was carried out (II). A region where cells do not completely cover the BNC was selected to better focus to cellular extensions. Especially in the zoomed image below, filopodia and lamellipodia structures are shown with high detail (red arrows) and give an impression of the tight cell-material interaction. The images also show that the cells arrange in multilayers as nuclei are partially overlapping (DAPI staining). Another observation was that cells are often present in tightly oriented arrangements with favoured rather than random directions (white arrows in II). More images of phalloidin and phalloidin/DAPI stained MSCs adherent to BNC can be found as Supplementary Figures. This observation suggests that BNC provides some level of cell guidance. Such areas with polarised and elongated cells were observed both at the cell-material interface and also within the multilayer (III).

### BNC stimulates collagen-I fibre formation by MSCs

**Figure 4:**
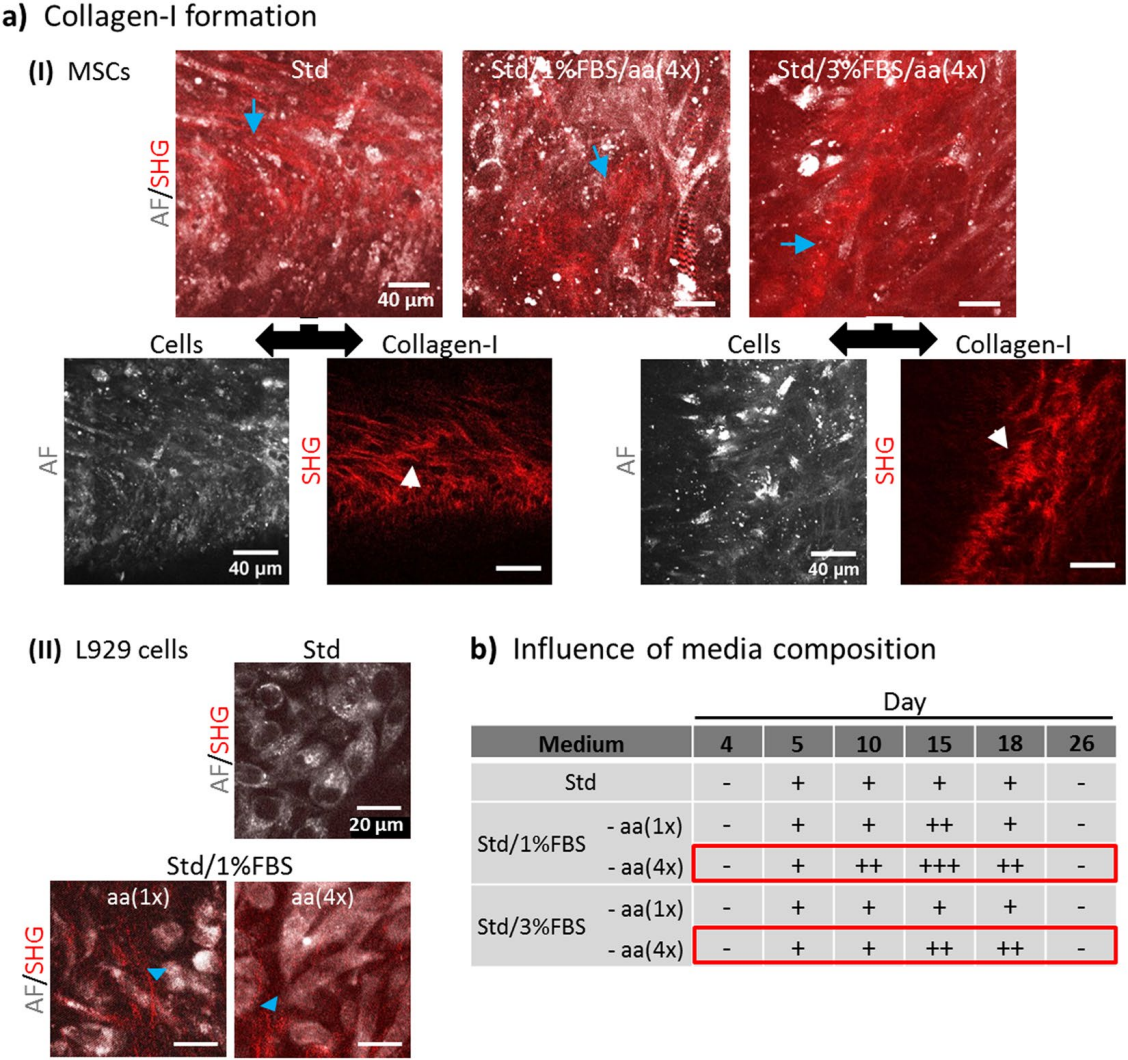
Collagen-I formation by cells seeded on BNC. (**a**) Two-colour multiphoton live cell images with AF (grey) showing the cells and SHG (red) representing cell-derived collagen-I. MSCs were cultured in various media of which standard growth (Std) and serum-reduced media (1% and 3% FBS) with elevated ascorbic acid (aa, 4x) resulted in collagen-I fibre formation (I, blue arrows). Images below (separated AF and SHG channel) reveal the collagen fibre structure and location relative to the cells. (II) Detection of collagen formed by L929 cells grown in Std and serum-reduced media. All images are representative for five individual experiments. (**b**) The table presents the time-frame of collagen-I formation by MSCs with various stimulation media. SHG signals were imaged using identical laser excitation and detection conditions. Analysis was performed semi-quantitatively by defining three categories of intensity (+, ++, +++).

**Figure 5:**
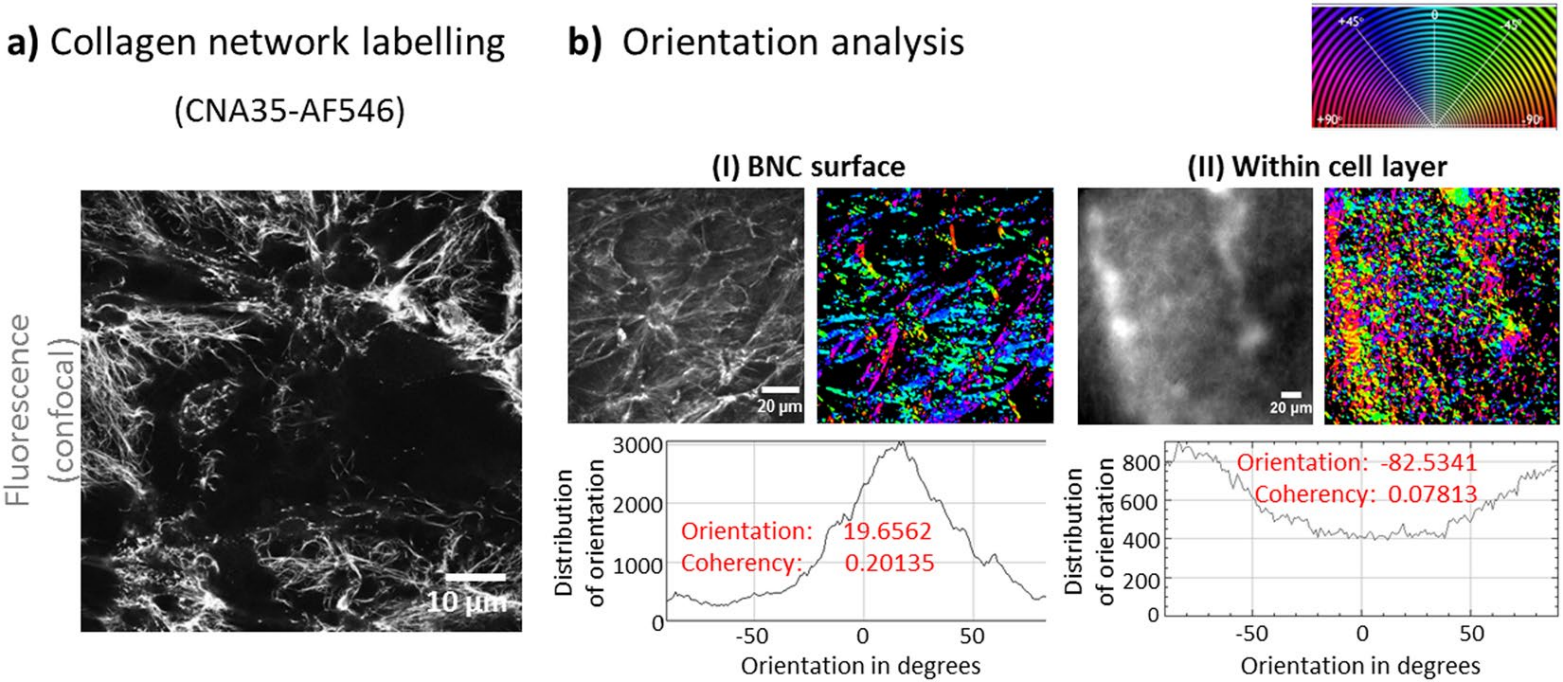
Collagen-I labelling and network analysis. (**a**) Collagen-I networks were independently imaged by confocal fluorescence microscopy (CFM) after specific staining with CNA35-AlexaFluor546 collagen-I binding protein. Much more details of fibre network structure compared to MPM images are visible (M = 20x). (**b**) Comparison of collagen fibre networks at the cell-BNC interface (I) and in between cells in a multilayer (II) using a fluorescence image and the corresponding S-colour survey image (colour code expressed by colour wheel at top right). Histograms mapping the incidence of fibre orientations from −90° to +90° including values for Orientation and Coherency are shown below.

From our previous studies, we were aware that MSCs are capable of producing extracellular matrix (ECM) collagen-I fibrous networks^40^ known to provide mechanical stability to tissues. However, collagen network formation (as detected by SHG) was dependent on how cells were cultured (3D largely superior over 2D culture). We also noticed that reducing serum content (FBS, fetal bovine serum) had a positive effect on collagen-I production as it forced cells to slow down or stop proliferation and by this, promoted ECM and tissue formation. A supplement known to have a positive effect on collagen formation is L-ascorbic acid (aa, also known as vitamin C), an important co-enzyme for biosynthesis and assembly of collagen fibres^51–53^. Here, we chose L929 as reference cells as they are well-studied connective tissue fibroblasts known to produce collagen-I stimulated by reduced serum and aa supplement^54^. With our cell seeding protocol, MSCs adhered and proliferated very well on BNC resulting in cell multilayers of up to 50 µm over the course two weeks. Apart from standard growth medium (Std), various stimulation media were tested for their capability to promote cells to produce collagen-I fibre networks. We modified Std medium by adding 25 and 100 µg ml^−1^ aa (1x and 4x) and reducing serum content from 20% to 3% or 1% (Fig. 4a). Such modified media appear to represent a strong trigger for the production of advanced artificial tissues. With all media tested, MSCs produced SHG-detectable collagen-I arranging into fibrous networks after 15 days (I, blue arrows). The collagen was present in between elongated multi-layered cells which had a flat morphology with long cell extensions (filopodia). However, detectable collagen was only present in certain zones within the cell layers as visible in the single-channel AF/SHG images below (white arrows). As expected, L929 cells formed the collagen in serum-reduced media (Std/1%FBS with 1x and 4x aa) but, unlike MSCs, not in Std medium (Fig. 4a-II, blue arrow heads). Collagen quantities formed by MSCs appear to be higher compared to those derived from L929. A three-dimensional representation of the formed collagen networks can be found for MSCs grown in Std/3%FBS/aa(4×) medium in Video 5. We were interested in comparing the amounts of SHG-detected collagen-I between media conditions in a time-dependent manner. The table in Fig. 4b provides a summary of the findings over 26 days after cell seeding. We decided to apply a semi-quantitative approach using 4 categories (−, +, ++, +++) to qualitatively classify the amount of 3D distributed collagen. The results show that collagen-I formation in all conditions began with day 5 and was still present at day 18. Different extents and time-frames became obvious with serum reduced conditions (1%/3%) and elevated levels of aa (4×, 100 µg ml^−1^) being the strongest stimuli (red-lined boxes). Compared to the other conditions with this combination of media constituents, collagen amounts were still increasing after day 10 with markedly higher overall amounts at days 15 and 18. These findings emphasise that serum depletion combined with increased aa induce stronger ECM production compared to Std medium. Next, we studied the collagen-I networks in more detail. SHG primarily detects highly assembled collagen fibres and fibre bundles, but much less thinner fibre networks, thus excellent results are possible particularly with native tissues. To better judge the morphology of collagen network formed by MSCs seeded on BNC, we chose to detect the collagen using CNA35, a highly specific collagen-I binding protein, labelled with a common fluorescence marker. Figure 5a shows highly resolved images of the fine structure of individual fibres at the cell-BNC interface with the morphology of native collagen-I networks (MSCs for 3 days on BNC, medium: Std). An interesting question was if collagen network structures would differ between the cell-BNC interface and in between cells. To answer this question, we chose BNC samples 3 days and 10 days after seeding with MSCs. In the 3 day sample, only a cell monolayer had formed whereas after 10 days, several layers of MSCs were seen. The BNC-cell constructs were collagen-I-labelled with CNA35-AF546 and imaged (Fig. 5b-I and -II). To compare collagen-I networks, we performed an orientation analysis as previously applied^40^ (histograms) and determined the fibre angle distribution at the material surface (I) with the situation within the cell layer (II). The relationship of fibre angle distribution is expressed by the values for ‘Orientation‘ and ‘Coherency‘ which are computed with Dominant Direction, a function in the plugin Orientation J (Image J). The most frequent or dominant orientation (angle in degrees) refers to the peak maximum in the histograms. The ‘Coherency‘ value (values from 0 to 1) is a measure for peak width and therefore expresses how dominant the maximum orientation is. A ‘Coherency‘ of 1 refers to a very sharp peak whereas with a value close to 0 the distribution is very broad. The histograms reveal that compared to the situation further away from the surface (within cell multilayer, II) the fibre angle distribution directly at the cell-material interface (I) was narrow (~0.201 vs ~0.078), so a much higher degree of orientation was present at the surface. These experiments were performed in triplicates and images at various distances from the material surface were analysed (image stacks).

### Growth on BNC alters the cell differentiation pattern of MSCs

**Figure 6:**
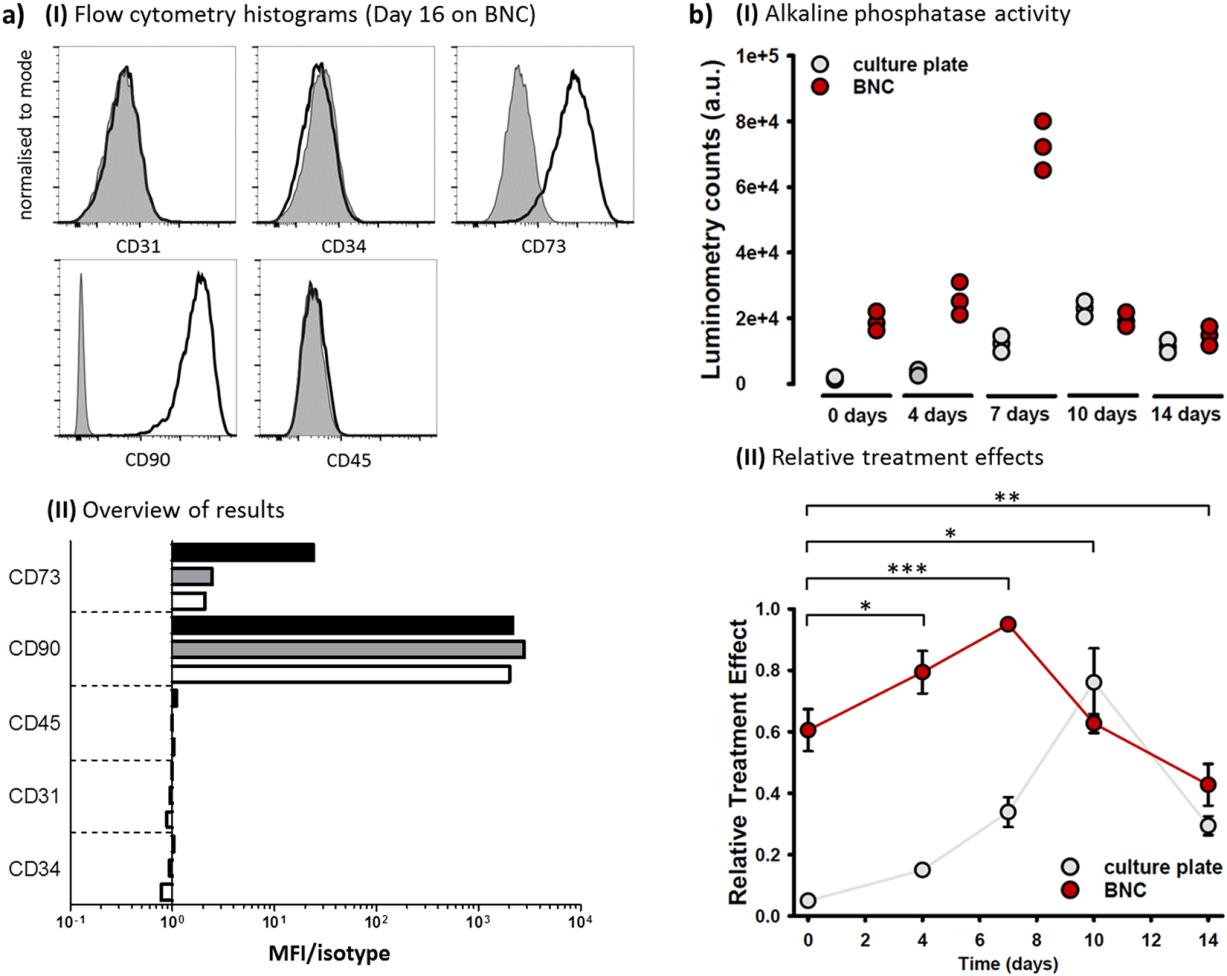
Differentiation status of MSCs. (**a**) MSCs were cultured on BNC or culture plate for 16 days and analysed for expression of CD31, CD34, CD45, CD73, and CD90 by flow cytometry. (I) Overlays show expression of indicated molecules (black) and corresponding isotype controls (grey, filled) at day 16 on BNC. (II) Differentiation of MSCs was analysed at day 0 (2 days after starting cell culture (white bars) and after 16 days on plastic plates (grey bars) or BNC (black bars). Graph represents mean fluorescence intensity (MFI) of surface markers relative to their isotype controls. (**b**) (I) AP activity (luminometry) data from MSCs seeded on BNC compared to standard cell culture plates (control) are displayed versus days after seeding. In (II), relative treatment effects, calculated based on the non-parametric model, reveal a significantly enhanced AP activity with major differences up to day 7, at which point activity already decreases for cells growing on BNC, while a similar effect takes up to day 10 for culture plate growing cells. ‘*’ Signs indicate time accumulating group differences between cells on BNC and culture plate and denote significances as follows: *p < 0.05; **p < 0.005; ***p < 0.0005.

Another important question to answer was ‘does growth on BNC alter the differentiation status of MSCs’ which could affect collagen-I formation. We tackled this question with a dual approach, (i) performing flow cytometry to test for cell identity using an accepted set of MSC markers and (ii) testing osteogenic differentiation using an AP assay. In flow cytometry, we analysed cell differentiation of MSCs 2 days after starting their culture and after 16 days on BNC versus standard cell culture plastic surfaces (Fig. 6a). Purity and differentiation of MSCs were tested by immunostaining with specific antibodies to the following cell surface markers: CD31, CD34, CD45, CD73 and CD90. The phenotype definition of MSCs requires expression of both, CD73 and CD90, together with a lack of expression of a leukocyte-specific marker (CD45), of a hematopoietic progenitor and endothelial cell marker (CD34)^55^, and of a cell marker for circulating cells (CD31)^56^. Histogram overlays for cells after 16 days on BNC are shown in Fig. 6a (upper part; histograms of day 0 and day 16 on plastic can be found in Supplemental Materials). The graph below demonstrates Mean Fluorescence Intensity (MFI) of CD markers of all cell samples relative to their isotype controls. It becomes obvious that the MSCs do not change their expression profile for CD31, CD34, CD45 and CD90 during the 2 weeks culture independent of the growth surface. This shows that there was no significant contamination with other or differentiated cells both before and after the culture period. However, the marker CD73 increases specifically on BNC. At day 0, the marker was detected 100% positive, but only at a low expression level. At day 16, the expression level was still only weakly positive on plastic, but around 10x stronger on BNC.

The results of the AP assay (MSCs on BNC versus plastic plates) are shown in Fig. 6b. All tests were performed with an identical cell number (8,000 cells). A comparison based on protein amounts (BCA protein quantitation assay) was not possible due to remaining serum from growth medium inside the BNC fleeces after washing steps, which would have interfered with the test. Lysis buffer with CSPD substrate (negative control) created almost no luminometry counts (mean ± s.d.: 32 ± 5), whereas MG-63 cells (cell control, pre-cultivated for 1 week) showed positive results (mean ± s.d.: 8638 ± 1025), as expected. The plot in (I) displays the variation in the samples over time (14 days). On the control surface (culture plate) luminosity increased over time and reached a maximum at day 10 with a value almost 3 × higher than in MG63 cells. At day 14, it decreased to the level of day 7. AP activity of MSCs grown on BNC was much higher with a maximum value already at day 7. After that, luminosity levels equal that of cells on culture plates. Calculated relative treatment effects along with the pointwise 95% confidence intervals are shown in the graph below (II). Like the previous observation, RTEs increase in both up to day 7, indicative of improving cell activity, with cells growing on BNC outperforming those cultured on standard culture plates. Statistical analysis showed significant accumulating differences between both groups with progressing time (up to day 4: P = 0.0202, up to day 7: P = 0.0003, up to day 10: P = 0.0068, up to day 14: P = 0.0038) and further revealed significant changes with time during the course of the experiment (up to day 14: P < 0.0001). Moreover it seems that different substrates not only influence group-related changes, but also the fashion their activity increases with time up to day 7, meaning that the time profiles of the two groups are not parallel and the hypothesis of ‘no interaction between treatment and time’ can be accepted (up to day 7: P = 0.8669810). Yet, this effect did not prolong to day 14 (P = 2.564107e-18), since AP activity decreased similarly for BNC and culture plate grown cells at day 7 and 10, respectively.

## Discussion

Our results underline the possibilities of MPM in the imaging of biomaterials with an AF or SHG signal response and the study of cell anchoring and ingrowth into pore structures. In our investigations, we observed that BNC, purified by means of the standard alkaline purification method, still exhibits an AF signature. An explanation for the AF signal would be remaining protein structures from the bacteria forming the BNC or from the protein containing culture medium, although the material was extensively washed and treated with harsh chemicals. Whereas BNC can be further purified to meet the thresholds for pro-inflammatory bacterial lipopolysaccharides (LPS), a required quality control step for medicinal products, here, the remaining protein content served the purpose of the study. Obviously, after alkaline purification, there are remaining autofluorescent biomolecules left that attach very strong within the BNC network. We observed no AF pattern in the dried state, but some SHG signal distributed in large dots. This may be due to crystalline regions in the material causing SHG to occur, because SHG depends on crystalline structures. Results show that some hydration was required for AF and SHG to occur, which happened very fast. When comparing these developing AF and SHG patterns, it becomes obvious that it is not the fibres that show AF, but some curly structure that changes into dots in the fully hydrated material which requires time for the water to penetrate the fibre network. Looking at the SEM image in Fig. 1a-III, it becomes clear that BNC is not consisting of more or less isolated fibres and bundles. Instead, there are many cross-connections between the fibre bundles that build up the porous structure, and these interconnections are likely surrounded by proteinaceous structures containing autofluorescent amino acids. These structures cause the AF signals which reorganise in the course of ongoing hydration finally leading to the dots. This also fits well with our observation that with BNC derived from alternative bacterial strains with a different cell size, we observed more elliptic dot patterns. Reappearance of the full pattern seen in hydrated BNC was very slow which was likely due to the BNC being rolled and therefore, densely packed with restricted availability of fibres for hydration. Only increase of rehydration temperatures or better testing of non-rolled BNC would help to study the origin of AF structures further. Low distance z-stacks of AF/SHG images and later 3D reconstructions would be required for this. The BNC fibres themselves slightly changed their morphology during hydration, as well as they were thinner directly after initial contact with water (Fig. 1b-I) compared to the fully hydrated state (Fig. 1b-II, lower left). Here, no change was seen in the BNC from the alternative strain.

As mentioned, BNC is gradually built up by microorganisms and formed at the interface between air and medium. From previous SEM studies, it was well documented that the fleeces can be differentiated into a top, middle and bottom layer^57^. Newly formed fibres exposed to air partially stick together leading to differences in network structure and surface porosity compared to the bottom side^18^. Within the fleeces, bacteria are surrounded by the nanocellulose network with varying nutrient and oxygen supply. Our results showed that channels of various widths are present below the surface and point towards multiple directions (Fig. 1). Most of these channels transverse the material, but some also run parallel to the surface. We suggest that nutrient supply occurs through these channels. Our MPM results further confirmed that BNC is not a homogenous, isotropic material, but is built up from parallel fibre sheets running in various directions which frequently change their orientation. We also detected structural variations with BNC fibres of varied thickness, degree of bundling and organisation.

Cell-material interaction takes place at the surface, so we studied this aspect in more detail. We did not observe an even surface, but lacerations (around 20 µm in size) and cracks all over, which in total largely increase the surface area. When focussing from outside into the material, it was the AF pattern that always appeared first. Given that AF derives mainly from proteins excreted by cells, we conclude that it is likely that lacerations/cavities are left behind after bacteria aggregations are washed out from the fleeces during their purification. The cracks may be formed by mechanical tensions and were found to be large enough for cells to grow into as niches. Islet structures (Fig. 2a-II) in which SHG and AF patterns are different may be identical to cavity structures which were observed using phase contrast imaging. Altogether, cells should be able to settle and anchor much better onto such a surface with many possibilities of cell-material interaction. They may act as environmental seeding core for MSCs. We observed that even though MSCs homogenously attached to the BNC surface, growth in multilayers (detected by 3D AF images) only started in local spots. The same was observed for collagen-I fibre networks that were detectable (beginning at around day 5) in local zones before being formed all over the surface. We believe that this may be due to the presence of isolated surface cavities in which the cells meet ideal conditions for anchoring and proliferation, which suggests the importance of these structures.

MSCs were selected in this study because they are widely used in TE and are known to produce collagen-I. Due to this fact, they usually adhere well on various materials. Cells requiring specific sets of matrix proteins for adhesion, like epithelial cells, have been shown to adhere less well (not shown) and thus, require surface modifications and/or coating in order to adhere to BNC. We have observed an excellent adhesion capacity on the BNC which, in addition, may partially derive from their long and thin filopodia that are ideal for anchoring to or growing into even tiny cracks on the surface (Fig. 3b). This is supported by their very strong attachment behaviour which required extended trypsin treatment even after removal of serum from within the BNC (flow cytometry and AP assay results). Imaging of cytoskeleton staining with phalloidin and high-intensity cellular AF multiphoton imaging shed light into how cells anchor via a large number of (in part very long) cell extensions to the uneven surface of BNC. We have also observed cell regions with clear orientation patterns close to the surface and further above (Fig. 3b-II/III). Polarised structures are found in tendon and ligament, bone and muscle tissue, and their engineering is supported by dynamic cultivation and/or mechanical treatment like stretching. Control of material surface micro-structures (e.g. with guided-assembly lithography^58^) can facilitate the engineering of such structures. Thus, it would be interesting to test if and how orientation of MSCs on BNC would be influenced by such surface modification or by stimulation of cell differentiation or media supplements. The observed cell orientation under static culture conditions with unprocessed BNC suggests that this material may be suitable for the engineering of such specialised tissues requiring cell polarisation.

SHG imaging was used to also detect native collagen-I fibres in 3D, and it was possible to differentiate it from BNC fibres, because other than BNC collagen-I is located in the proximity of the cells). Unlike on plastic cell culture dishes, collagen-I was already formed when cells grew as a 2D monolayer at the cell-material interface^40^. Stimulated by the BNC surface, MSCs produced collagen-I networks comparable to culture in cell spheroids where they experience a physiological 3D micro-environment as shown in our previous study^40^. Collagen on the BNC scaffolds, however, was present for markedly longer periods (18 vs 12 days). In addition, in the above mentioned study, L929 did not form collagen-I in 2D and in 3D cell spheroids but on BNC thus, suggesting that BNC has an inherent stimulatory effect also to these cells. The comparison of different stimulation media on collagen-I formation showed that was mainly higher concentrations of ascorbic acid that triggered elevated collagen-I levels. This can be explained by the fundamental role of this substance in collagen biosynthesis, modification and assembly.

Alternative imaging of collagen-I networks using CNA35-labelling produces much more detailed images, because the very fine fibres not susceptible to SHG are also mapped. The resulting fluorescence images prove that MSCs can form native, tissue-like complex fibre network arrangements on BNC scaffolds. Another advantage is that nanocellulose is not being detected which is helpful especially at the interface of cells and biomaterial as imaged here. In SHG imaging, in some cases, signal from collagen-I may be mixed up with BNC. This problem, however, can be resolved by differentiating signal from in between or close to cells (collagen-I fibres) from signal co-localising with AF dots from BNC. CNA35-based fluorescence labelling however, is an invasive technique and efficiently detects non-fibrous intracellular pro-collagen as well, so cell perforation caused by fixation should be avoided. Orientation analysis performed with images from CNA35-labelled collagen-I points towards much more orientated fibre networks directly on the surface compared to a much broader distribution and almost no favoured direction in the cell-cell environment with highly isotropic networks. This suggests that BNC has an instructive effect on MSCs’ ability to form collagen-I fibre networks. This is not very surprising as collagen networks at the interface fulfil a different function at the interface (attachment) than between cells (cell-cell-mechanical contact). Fibre orientation analysis in 3D was tested in our labs as well and was found to be possible with the use of CNA35 labelling, however, it requires very small z-distances between images to identify and reconstruct fibres running through multiple image planes. Quantitative methods for fibre thickness analysis in Image J were also tested. This method would be worthwhile testing for more detailed analyses of collagen network structure close to the cell-material interface. The results from CNA35 labelling also made clear that collagen-I networks were already detectable at day 3, whereas with SHG microscopy, they could be imaged only beginning from day 5 (Fig. 4b).

By using flow cytometry, we wanted to prove the identity of MSCs and potential differentiation effects of BNC on our cells. The results determined a cell population with high purity, low number of dead cells and a low degree of granularity both before and after the 16 days growth period. The large increase in the CD73 marker after growth on BNC suggests a specific stimulatory effect of this biomaterial to the MSCs. However, as MSCs grew very dense on the BNC fleece, it cannot be ruled out that a cell density effect influenced this result, at least in part. A comparison with 3D cell pellets and analysis at two or three intermediate time points (e.g. 3, 6 and 10 days) may help answer this question. CD73 is an MSC marker which catalyses the dephosphorylation of extracellular AMP into adenosine (ecto-5′-nucleotidase) that can enter the cells^59^. The cell surface protein (which is present in a variety of tissues) acts as a regulatory factor in the osteogenic differentiation of MSCs^60^. It was also reported that CD73 and CD90 were independent of passage number and time in culture^59^, a finding that would strengthen our theory that BNC stimulates MSC osteogenic differentiation. For more detailed studies of osteogenic differentiation, further analyses including the expression of RunX2 and Osteocalcin would make sense. Considering the increased and accelerated build-up of AP activity in MSCs grown on BNC compared to regular cell culture plates, it seems very likely that the used BNC surfaces stimulate osteogenic differentiation of MSCs. Major differences are particularly visible up to day 7. Decrease in AP activity in BNC-growing cells is visible already at day 10 compared to cells grown on culture plates (day 14).

## Conclusions

By applying a multiphoton imaging approach, we provided insights into BNC material surface and inner micro-structure, as well as beneficial effects on cell behaviour including the formation of collagen-I networks. This is of large impact to the field of TE and beyond, including regenerative medicine, orthopaedics/traumatology, dermatology as well as bioprocess engineering and all fields in which cell-biomaterial interactions are of major interest. It is well-documented that MSCs^61^ are capable of sensing their biochemical and biomechanical^62^ environment, including the proximity to other cells, which seems to be finely tuned for cells to form collagen networks, predominantly in zones of 3D cell arrangement. In this perspective, the correct cell-material and intercellular anchoring and ECM lattice formation represent the hallmark for tissue architecture definition and stabilisation^61^. Our results suggest that 3D organisation to multilayers plays a superior role for promoting collagen-I formation. The findings also prove that biomimetic surface micro-structures on BNC are by far more potent over simple 2D environments, probably by provision of a more natural biomechanical anchorage for cells. Future research will have to address cell-BNC interaction in more detail, including assessment of focal adhesion complexes to optimise cell adhesion stability. Another issue would be to perform quantitative fibre network analyses in order to optimise collagen networks^40^ for quality assessment purposes. Studying refined media compositions containing growth factors, like bone morphogenetic proteins (BMPs), would also be important. It will be likewise fundamental to determine the differentiation status of MSCs during their growth on BNC (using markers like Runx2 or ALP) and for bone TE to carry out osteogenic differentiation and compare results with osteoblasts. From the material point-of-view, more forms of BNC including 3D-patterned ones^58^, BNC fleeces with defined pore sizes, fibre density profiles and chemical surface modifications are accessible and would help to engineer tissues for advanced medical use.

BNC - as a cell-derived natural product – possesses micro- and nano-structured environments for MSCs which may support their physiological needs. To further explore specific effects of surface micro-structures on collagen network formation, functional properties may be transferred to BNC nano-fibre textures as recently shown by Bottan *et al*.^58^, using Guided Assembly-based Biolithography (GAB), a replica molding technology. Those authors have demonstrated effects on cell polarisation, surface coverage and proofed beneficial effects on skin regeneration.

## Electronic supplementary material

Supplementary Information

Video 1

Video 2

Video 3

Video 4

Video 5
