# Supplementary material for: Bacterial nanocellulose stimulates mesenchymal stem cell expansion and formation of stable collagen-I networks as a novel biomaterial in tissue engineering
Source: Sci Rep. 2018 Jun 20;8:9401. doi: 10.1038/s41598-018-27760-z (PMC6010428; doi:10.1038/s41598-018-27760-z)
Supplement: Supplementary file 1 — Supplementary Information [file 41598_2018_27760_MOESM1_ESM.pdf]

Supplementary Information

Bacterial nanocellulose stimulates mesenchymal stem cell expansion and formation of stable collagen-I networks as a novel biomaterial in tissue engineering

Martin Vielreicher[1,], Dana Kralisch[2], Simon Völkl[3], Fabian Sternal[1], Andreas Arkudas[4] & Oliver Friedrich[1]*

[1] Institute of Medical Biotechnology, Department of Chemical and Biological Engineering, Friedrich-Alexander University of Erlangen-Nürnberg, Paul-Gordan-Str. 3, Erlangen, 91052 (Germany)

[*] Corresponding author; E-mail: martin.vielreicher@mbt.uni-erlangen.de

[2] Institute of Pharmaceutical Technology. Faculty of Biology and Pharmacy, Friedrich-Schiller-University Jena, Lessingstr. 8, Jena, 07743 (Germany)

[3] Department of Internal Medicine 5, Hematology and Oncology, University Hospital Erlangen, Ulmenweg 18, 91054 Erlangen (Germany)

[4] Department of Plastic and Hand Surgery, University Hospital Erlangen, Friedrich Alexander University of Erlangen–Nürnberg, Krankenhausstr. 12, 91054 Erlangen (Germany)

VIDEOS

All videos presented are 3D image stacks (.avi format, 2 frames per second).

Multiphoton excitation was at λ=810 nm, signal detection was either separated into a channel recording autofluorescence (AF, λ>460 nm) and a channel for Second Harmonic Generation signal channel (SHG, λ=405±20 nm) or the raw signal was detected by one channel (AF and SHG unseparated). Z represents the distance between images in z direction.

Video 1. BNC structure imaged with MPM. Imaging into the BNC hydropolymer material (15 images, z-distance: 1 µm). Left sequence: punctuate AF structures. Right sequence: fibrous structure of nanocellulose shown by SHG.

Video 2. Variations in BNC structure. The sequences (30 images, z: 1 µm) reveal irregularities in BNC structure both in the AF and the SHG channel likely derived from the materials natural origin (biotechnological production).

Video 3. Cavity structures on the BNC surface. BNC surface imaging (MPM, z: 0.5 µm, 23 images) focused on an islet structure with a characteristic agglomerate pattern: very intense AF signals, wavy appearance of SHG signal different to normal nanocellulose structure.

Video 4. Cell-BNC interface. A small distance images series (16 images, z: 0.5 µm) is shown uncovering the cell-material interface of MSCs attached to the surface of BNC (AF+SHG unseparated channels).

Video 5. Collagen formation by MSCs on BNC. A stack of 18 images (z-distance: 5 µm) depicts collagen-I networks within the cell multilayer (80 µm). Cells were grown in medium with reduced serum and elevated ascorbic acid (Std/3%FBS/aa(4x)) for a period of 15 days.

SUPPLEMENTARY DATASETS

Supplementary Dataset 1: Actin cytoskeleton staining. MSCs at the cell-material interface labelled with phalloidin-PF546 and DAPI or phalloidin-PF546 alone and imaged using an inverted epi-fluorescence microscope. Different regions are shown.

Supplementary Dataset 2: Flow cytometry histograms. MSCs after 16 days on cell culture plastic versus BNC.

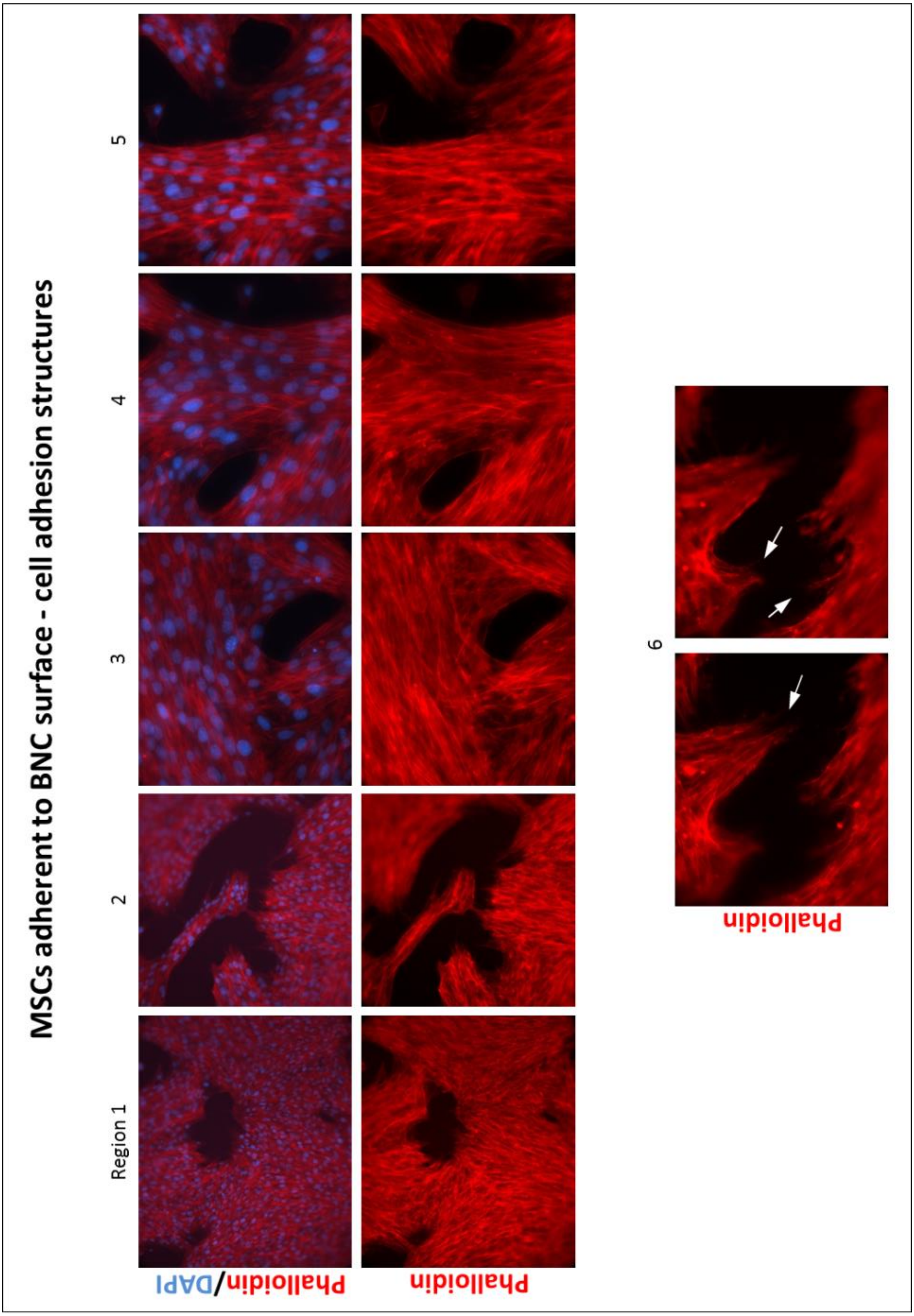

Supplementary Dataset 1: Actin cytoskeleton staining

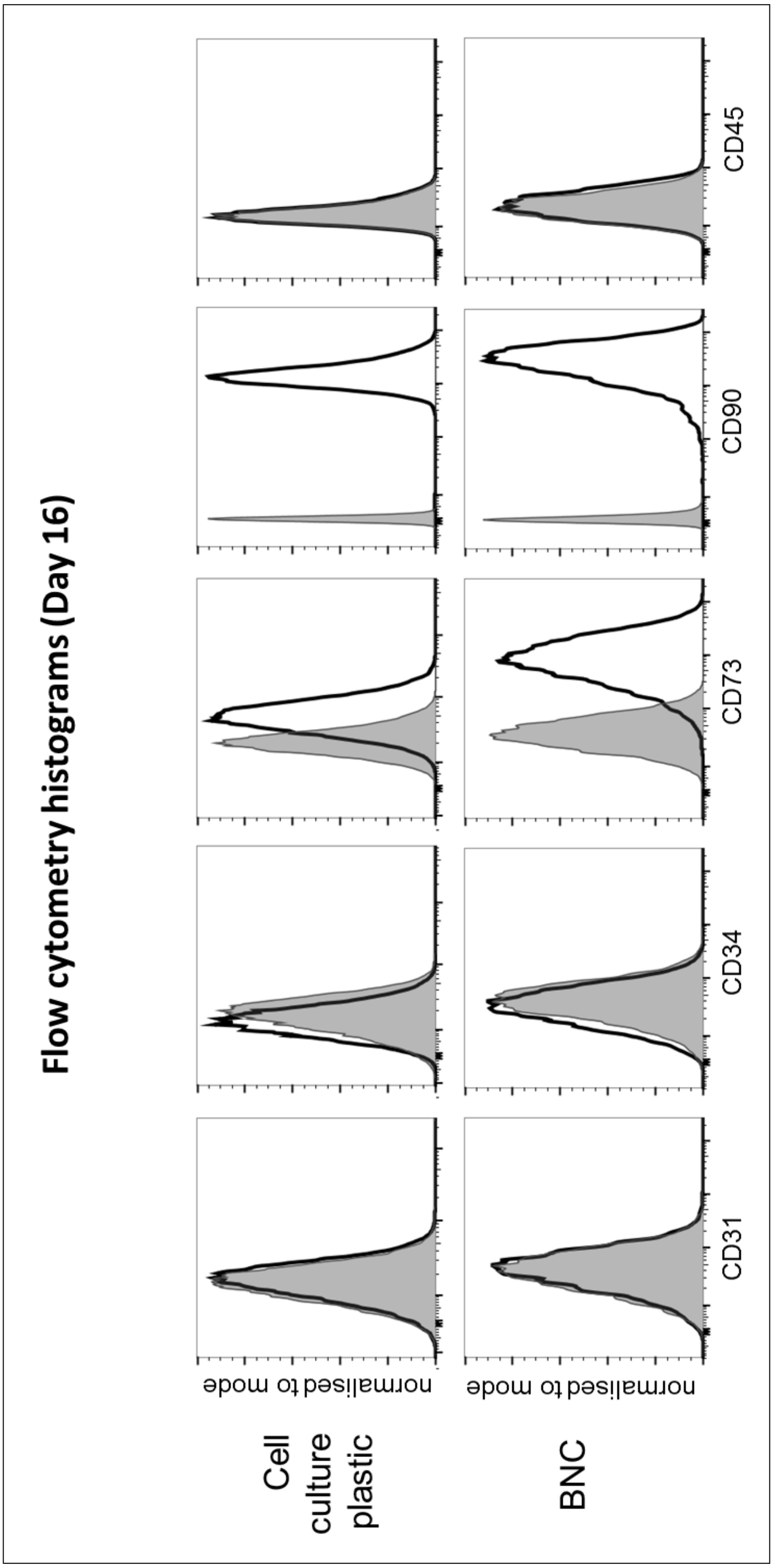

Supplementary Dataset 2: Flow cytometry histograms
